# Evolution of satellite DNA sequences in two tribes of Bovidae: A cautionary tale

**DOI:** 10.1590/S1415-475738420150094

**Published:** 2015

**Authors:** Mariella Nieddu, Roberto Mezzanotte, Giuseppina Pichiri, Pier Paolo Coni, Gian Luca Dedola, Maria Luisa Dettori, Michele Pazzola, Giuseppe Massimo Vacca, Renato Robledo

**Affiliations:** 1 Department of Biomedical Sciences, University of Cagliari, Monserrato, Italy; 2 Department of Surgical Sciences, University of Cagliari, Cagliari, Italy; 3 Department of Veterinary Medicine, University of Sassari, Sassari, Italy

**Keywords:** Bovidae, satellite DNA, fluorescence *in situ* hybridization, pericentromeric region

## Abstract

Two clones, Bt1 from *Bos taurus* and Om1 from *Ovis orientalis musimon*, were used as probes for hybridization on genomic DNA and on metaphase chromosomes in members of Bovini and Caprini tribes. Bt1 and Om1 are sequences respectively belonging to the 1.715 and 1.714 DNA satellite I families. Southern blots and fluorescence *in situ* hybridization experiments showed completely coherent results: the Bovini probe Bt1 hybridized only to members of the Bovini tribe and not to members of Caprini. Likewise, the Caprini probe Om1 hybridized only to members of the Caprini tribe and not to members of Bovini. Hybridization signals were detected in the heterochromatic regions of every acrocentric autosome, except for two pairs of autosomes from *Capra hircus* that did not show hybridization to probe Om1. No signal was detected on X and Y chromosomes or on bi-armed autosomes. Remarkably, probe Om1 showed almost 100% homology with a bacterial sequence reported in *Helicobacter pylori*.

## Introduction

The Bovidae, an important mammal family in the Artiodactyla order, includes a number of species such as cattle, sheep and goat, which are highly relevant from both economic and social viewpoints. Moreover, Bovidae is the most diverse family within the Artiodactyla order, and includes approximately 140 extant species (Nowak, 1999) that have been classified in several subfamilies, which, in turn, are further divided into tribes (Gentry, 1992; Gallagher Jr *et al*., 1999). However, the systematic and phylogenetic relationships among the various species are still under discussion.

Many studies have attempted to reconstruct phylogenesis in Artiodactyla by means of the analysis of endogenous retroviruses (Chessa *et al*., 2009), microsatellites (Vacca *et al*., 2011), gene sequences (Carcangiu *et al*., 2011) and mitochondrial DNA (Naderi *et al*., 2007; Achilli *et al*., 2009; Vacca *et al*., 2010; Lei *et al*., 2011; Meadows *et al*., 2011). Nevertheless, the systematic of the Bovidae remains controversial, because evolutionary steps such as rapid radiation and morphological convergence led to a highly variable chromosome number within this family, ranging from 30 to 60 (Chaves *et al*., 2005). Domestic cattle is considered to have the ancestral karyotype (2n = 60) due to the presence of telocentric chromosomes. However, rearrangements such as centric fusions between uniarmed chromosomes led to karyotype differences, *e.g*. the river buffalo (*B. bubalis bubalis*) with 2n = 50 and the swamp buffalo (*B. bubalis kerebau*), with 2n = 48 (Iannuzzi *et al*., 1996).

A significant portion of eukaryotic genomes is represented by repeated sequences, among which a large fraction includes sequences repeated in tandem, known as satellite DNAs (for a review, see Plohl *et al*., 2008), often located at or near the centromeres (D'Aiuto *et al*., 1997). A typical satellite DNA is composed of thousands of monomeric units, arrayed in tandem in head-to-tail configuration and located in constitutive heterochromatin (Plohl *et al*., 2008). Since no protein-coding function has been assigned to satellite DNAs to date, these sequences are not evolutionarily constrained, and therefore change more rapidly (Pita *et al*., 2009) than any other genomic sequences, making them useful for comparative studies. In this paper, we analyzed two satellite sequences, one obtained from *Bos taurus* and the other from *Ovis orientalis musimon*, in four members of the Bovinae and Caprinae subfamilies, respectively *Bos taurus*, *Bubalus bubalis*, *Ovis orientalis musimon* and *Capra hircus*. Our results provide support to the idea that the distribution and organization of DNA satellite sequences may provide an additional tool to help in resolving the still-controversial points involved in the systematics of Bovids.

## Material and Methods

### DNA samples and probes

Sampling included the following species: *Bos taurus*, *Bubalus bubalis*, *Ovis orientalis musimon* and *Capra hircus*. Peripheral blood samples were collected and DNA was extracted according to Cau *et al*. (1992). Genomic DNA from *Bos taurus* was digested to completion with restriction enzyme *Hae*III. Following gel electrophoresis, a discrete band of approximately 300 base pairs was isolated, purified and cloned in vector pJET1.2/blunt (Thermo Scientific). Likewise, DNA from *Ovis orientalis musimon* was digested with *Alu*I, and a discrete band of approximately 800 base pairs was isolated, purified and cloned in the same vector. Several clones were sequenced (Macrogen Europe, Amsterdam); two of them, Bt1 (from *Bos taurus* digested with *Hae*III) and Om1 (from *Ovis orientalis musimon* digested with *Alu*I), were used as probes for Southern and *in situ* hybridization.

### Chromosome preparation

Metaphase preparations were obtained following the procedures described in Moorhead *et al*. (1960). Briefly, heparinized lymphocytes were cultured for 72 h at 37 °C in RPMI-1640 medium (Invitrogen), supplemented with 10% fetal bovine serum (FBS), 1% penicillin and streptomycin, and 100 μL/mL phytohemoagglutinin (PHA). Cells were arrested at metaphase by adding colcemid (10 mg/mL) for 2 h. Hypotonic treatment was carried out with 0.075 M KCl for 10 min; cells were then fixed with 3:1 methanol:acetic acid for 30 min and spread onto clean slides.

### Fluorescence *in situ* hybridization

Slides containing chromosomes were treated with 70% formamide (in 2xSSC), pH 7, at 70 °C for 2 min and then dehydrated in an increasing ethanol series. Bt1 probe from *Bos taurus* DNA and Om1 probe from *Ovis orientalis musimon* DNA were labelled with Spectrum Green dNTP (Vysis) by nick translation (Nick Translation System BRL) and, after precipitation, they were dissolved in hybridization buffer (50% formamide, 10% dextran sulfate, 2xSSC, pH 7) to a final concentration of 20 ng/μL. Probes were then denatured at 70 °C for 10 min, put on ice for 5 min and hybridized overnight at 37 °C in a wet chamber. Following hybridization, slides were washed once in 2xSSC in 50% formamide for 5 min and twice in 2xSSC for 3 min. Slides were finally counterstained with 1 μg/mL propidium iodide (Sigma Aldrich). Results were evaluated with a digital image analysis system consisting of an epifluorescence Nikon Optiphot microscope and charge-coupled device camera (COHU) interfaced to the CytoVision system, version 2.7 (Applied Imaging).

## Results

### Sequencing and Southern blotting

**Figure 1:**
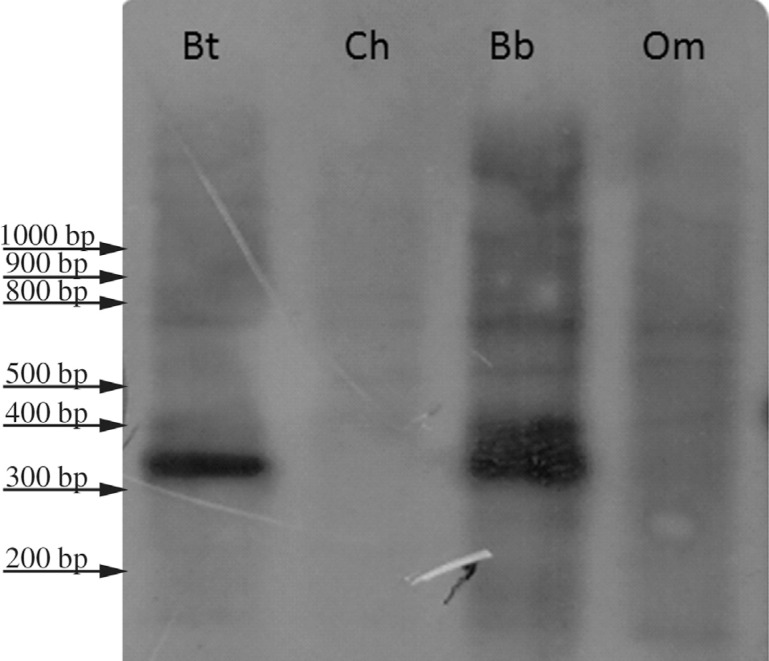
*Bos taurus* genomic DNA digested with *Hae*III and hybridized to probe Bt1. Signals are detected in genera *Bos* and *Bison*, but not in genera *Ovis* and *Capra*. Bt = *Bos taurus*, Ch = *Capra hircus*, Bb = *Bubalus bubalis*, Om = *Ovis orientalis musimon*.

Probe Bt1, isolated from *Bos taurus*, was 344 base pairs long (GenBank accession number: KM272302). The sequence, analyzed with BLAST (Altschul *et al*., 1997), showed very high homology (ranging from 99% to 93%) with several bovine satellite DNA sequences belonging to the 1.715 satellite I family. Likewise, homology of 94% and 83% was detected with DNA satellite I sequences of *Bison bonasus* and *Bubalus bubalis*, respectively. Accordingly, in Southern blot experiments, probe Bt1 gave strong positive signals when hybridized to *Bos taurus* and *Bubalus bubalis* genomic DNA, whereas no bands were detected when the probe was hybridized to *Ovis orientalis musimon* or to *Capra hircus* genomic DNA (Figure 1).

**Figure 2:**
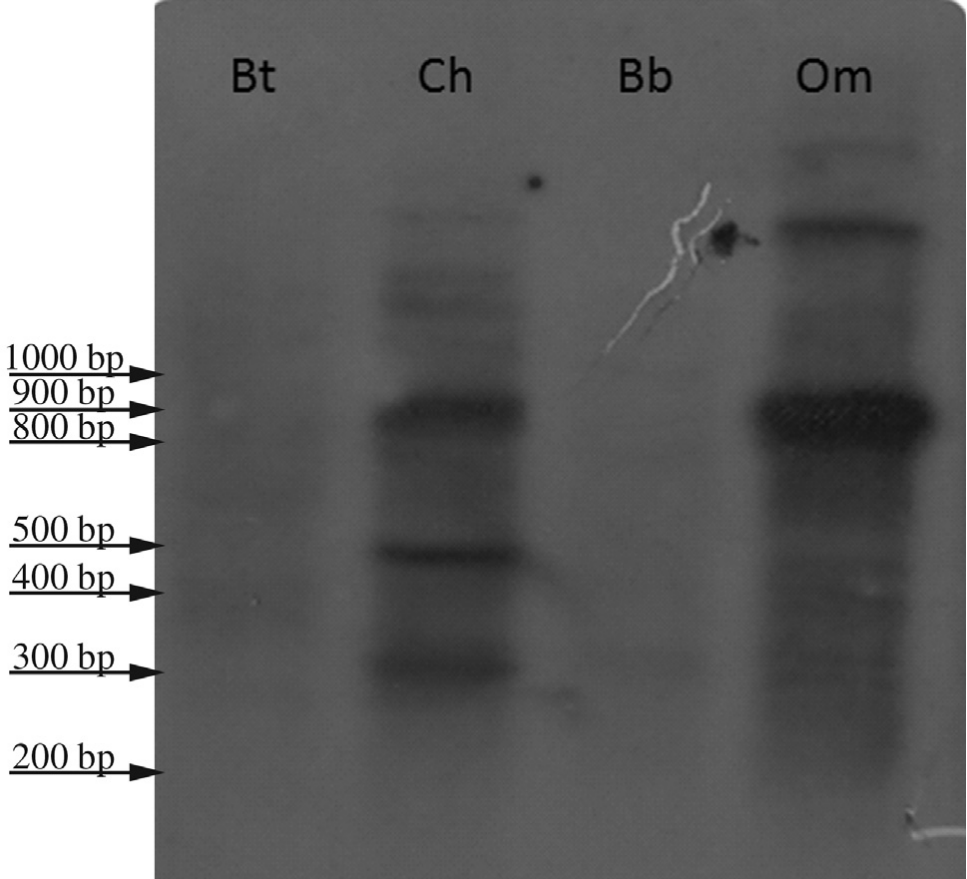
*Ovis orientalis musimon* genomic DNA digested with *Alu*I and hybridized to probe Om1. Signals are detected in genera *Ovis* and *Capra*, but not in genera *Bos* and *Bison*. Abbreviations as in Figure 1.

Probe Om1, isolated from *Ovis orientalis musimon*, was 816 base pairs long (GenBank accession number: KM272303). BLAST analysis showed that the sequence is nearly identical to satellite DNA sequences belonging to the 1.714 satellite I family present in *Ovis aries* and *Ovis amon*, and has high homology (87%) with a similar satellite DNA sequence present in *Capra hircus*. When hybridized to genomic DNAs, the Om1 probe gave positive signals in *Ovis orientalis musimon* and *Capra hircus*, whereas no signal was detected when the probe was tested with *Bos taurus* and *Bubalus bubalis* genomic DNA (Figure 2). Surprisingly, BLAST analysis showed that probe Om1 has a very high degree of homology, *i.e*. almost 100%, with a bacterial sequence reported in *Helicobacter pylori*.

### Fluorescence in situ hybridization

**Figure 3:**
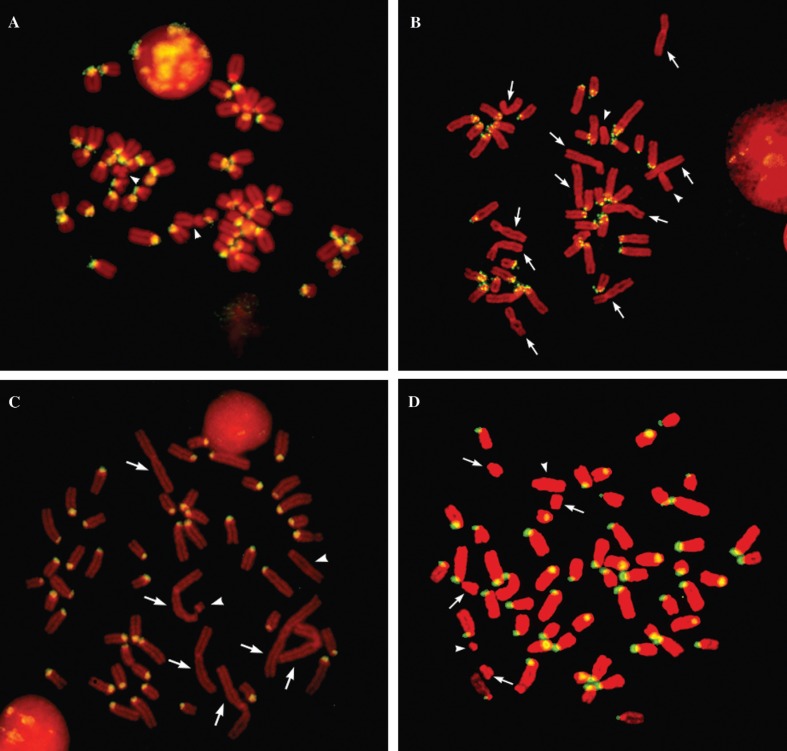
Fluorescence *in situ* hybridization. A) Chromosomes from *Bos taurus* hybridized to probe Bt1. Signals are present in pericentromeric regions of every autosome, but not on the X and Y chromosomes (indicated by arrowheads). B) Chromosomes from *Bubalus bubalis* hybridized to probe Bt1. Signals are present in pericentromeric regions of acrocentric autosomes, but not on the five pairs of bi-armed autosomes (arrows), or on the X chromosomes (arrowheads). C) Chromosomes from *Ovis orientalis musimon* hybridized to probe Om1. Signals are detected on all acrocentric autosomes. No signal was detected on the three pairs of bi-armed autosomes (arrows), or on the X and Y chromosomes (arrowheads). D) Chromosomes from *Capra hircus* hybridized to probe Om1. Signals are detected on acrocentric autosomes, with the exception of two pairs of autosomes (arrows). No signal is visible on the X and Y chromosomes (arrowheads).

The same probes were used for *in situ* hybridization on metaphase chromosomes. The Bt1 probe gave a positive signal on all pericentromeric regions of the acrocentric autosomes of *Bos taurus*, while no signal was detected on the X or the Y chromosome (Figure 3A). The same probe gave a positive signal on pericentromeric regions of all acrocentric autosomes of *Bubalus bubalis*, whereas no signal was observed in the ten bi-armed chromosomes or on the X and Y chromosomes (Figure 3B). No signal was detected on any chromosome of *Ovis orientalis musimon* and *Capra hircus* (data not shown).

The Om1 probe gave a positive signal on pericentromeric regions of all acrocentric chromosomes of *Ovis orientalis musimon*, but we detected no signal on bi-armed autosomes or on the X and Y chromosomes (Figure 3C). Exactly the same pattern was detected in chromosomes of *Ovis aries* (data not shown). In *Capra hircus*, a slightly different pattern was observed, with two additional pairs of autosomes giving no signal (Figure 3D). Finally, the Om1 probe did not hybridize on *Bos taurus* or *Bubalus bubalis* chromosomes (data not shown).

## Discussion

We analyzed two DNA satellite I sequences in different species belonging to the Bovidae family. The two sequences, Bt1 and Om1, were nearly identical to previously-described sequences reported as belonging respectively to the 1.715 and 1.714 DNA satellite I families (Chaves *et al*., 2005). When used as probes in Southern blots or in FISH, Bt1 and Om1 showed completely coherent results. Indeed, the Bt1 sequence hybridized to *Bos taurus* and *Bubalus bubalis* chromosomes and genomic DNA, while no signal was detected in *Capra hircus* and *Ovis orientalis musimon*. Accordingly, the Om1 sequence hybridized only to *Capra hircus* and *Ovis orientalis musimon* chromosomes and genomic DNA, but not to *Bos taurus* and *Bubalus bubalis*.

Similar sequences were analyzed in previous studies, though with conflicting results: Chaves *et al*. (2005) found that probe pBtKB5, from cattle, which shows 97% identity with probe Bt1, showed a positive signal in FISH experiments carried out on Caprini (genera *Ovis* and *Capra*). Other authors (Kopecna *et al*., 2012) found that probe BTREP15, from cattle, which shows 98% identity with probe Bt1, gave no signals when hybridized to chromosomes of Caprini tribes (genera *Ovis*, *Capra* and *Ammotragus*), in agreement with our results. However, the same authors detected hybridization signals when washing was done using lower stringency conditions (Kopecna *et al*., 2012). The cause of the discrepancy may be related to different lineages used, each having distinct repetitive sequences. Moreover, differences in hybridization stringency and between probes may also be involved. Although belonging to the same family of satellite I DNA, different DNA sequences were employed in the various studies, thus implying that the 1.715 family of satellite I is composed of a number of subfamily sequences not necessarily identical to each other. In particular, Bt1 probe used in the present work represents a shorter (344 bp) sequence that is included in the larger probes pBtKB5 (566 bp) and BTREP15 (581 bp) used in the above-mentioned studies. Kopecna *et al*. (2014) extended their analysis employing different satellite DNA clones isolated by laser microdissection of centromeric regions in 38 bovid species. In all species analyzed, the satellite I probe gave a strong signal on acrocentric autosomes and a much weaker, or no signal, on biarmed autosomes. Biarmed X chromosomes also showed no satellite I hybridization, while all acrocentric X chromosomes, with exception of those of Caprini, were positive (Kopecna *et al*., 2014).

DNA satellite I is located mainly at or near centromeres (D'Aiuto *et al*., 1997). As expected, sequences were detected on every acrocentric autosome. The lack of signal in the bi-armed chromosomes of *Bubalus bubalis* and *Ovis orientalis musimon* is probably due to the loss of heterochromatin following Robertsonian translocations, an event producing a change in the number of chromosomes but not chromosome arms (Robertson, 1916; reviewed in Robinson and Ropiquet, 2011). Our results agree with data produced by Chaves *et al*. (2000) and by Davila-Rodriguez *et al*. (2009), who found that the amount of constitutive heterochromatin is greater in all pericentromeric regions of acrocentric chromosomes than in metacentric or sex chromosomes. However, the lack of signal on two pairs of autosomes in *Capra hircus* suggests, as an alternative interpretation, that the loss or rearrangement of heterochromatin precedes or is independent of chromosome fusions.

Moreover, finding the same hybridization pattern shared by *Ovis orientalis musimon* and *Ovis aries*, with a slightly different pattern observed in *Capra hircus*, is consistent with current phylogeny. The lack of signal on X and Y chromosomes, which has also been reported previously (Chaves *et al*., 2000, 2005), may have alternative explanations: either the absence of highly repetitive DNA in sex chromosomes, as suggested by Hornaday *et al*. (1994), or higher evolution rates of these sequences due to the lack of homologous recombination in the male sex. The latter interpretation is supported by the presence of satellite DNA (1.715 family) detected using probe BTREP15 on the *Bubalus bubalis* X chromosome, but absent in *Bos taurus* and *Bison bonasus* (Kopecna *et al*., 2012, 2014).

Finally, a totally unexpected finding was the nearly 100% homology between probe Om1 (1.714 family of satellite DNA) with a prokaryotic sequence present in *Helicobacter pylori*, a Gram-negative bacterium involved in the etiology of peptic ulcer. Lateral transfer of bacterial DNA into a mammalian genome has been previously suggested (Salzberg *et al*., 2001), but never proven. A more likely explanation is contamination from other species, which may masquerade as lateral gene transfer (Willerslev *et al*., 2002). A clear case of cross-species contamination, where *Neisseria gonorrhoeae* contained multiple sequences derived from cow and sheep genomes, has been recently reported (Merchant *et al*., 2014).

In conclusion, our results support the idea that rapidly-evolving satellite DNAs may be a useful tool in the still-controversial systematic studies of Bovidae. Being able to differentiate at a subfamily or tribal level, they will provide the additional information necessary to clarify the phylogeny of that family. However, results must be interpreted with great caution, since differences in satellite DNA may be quantitative (variation in copy number) or qualitative (variation in nucleotide sequence). Identical probes, as well as identical experimental conditions, must be employed for obtaining comparable data.
